# Estimating the cost of chronic kidney disease in Australia

**DOI:** 10.1186/s12913-024-11953-6

**Published:** 2024-11-26

**Authors:** Sean Randall, Crystal M. Y. Lee, Elizabeth Thomas, Aron Chakera, Kevin E. K. Chai, Richard Varhol, Kanika Mehta, Ashley Irish, Johan Conradie, Narelle Hadlow, Delia Hendrie, James H. Boyd, Suzanne Robinson

**Affiliations:** 1 Deakin Health Economics, Institute for Health Transformation, Deakin University, Burwood Hwy, Burwood, VIC 3125 Australia; 2 School of Population Health, Curtin University, Perth, WA Australia; 3 Sir Charles Gairdner Hospital, Nedlands, WA Australia; 4 Baker Heart and Diabetes Institute, Victoria, Australia; 5 WA Country Health Service, Perth, WA Australia; 6 Western Diagnostic Pathology, Perth, WA Australia; 7 PathWest Laboratory Medicine, Perth, WA Australia; 8 Department of Public Health, La Trobe University, Victoria, Australia

**Keywords:** Chronic kidney disease, healthcare costs, Economic burden, Australia, Healthcare expenditure, Renal disease

## Abstract

**Introduction:**

Chronic kidney disease (CKD) is a significant burden on health systems globally, with limited up-to-date information on health system costs, particularly for non-dialysis patients. This study estimates the direct healthcare costs of CKD within Australia.

**Methods:**

The study utilised the CKD.WA dataset, a linked repository for the state of Western Australia, containing public and private pathology, hospital, emergency and mortality data for over 2 million people, along with a secondary dataset of general practice records. Costs were calculated for individuals with CKD in 2019 and compared to controls without CKD to identify costs attributable to CKD. Cost items included hospital, emergency, medication, general practice, pathology, dialysis and outpatient services. Costs were expressed in 2023 AUD.

**Results:**

There were 114,899 individuals with CKD in 2019. Average yearly costs attributable to CKD were $3,367 for Stage 1, $4,114 for Stage 2, $3,607 for Stage 3a, $6,572 for Stage 3b, $11,456 for Stage 4 and $62,558 for Stage 5. Non-dialysis hospital costs were the biggest contributor, followed by dialysis costs. The estimated total cost of CKD to Australia was $8.3 billion for 2019.

**Conclusion:**

These findings highlight the significant cost burden of CKD. While CKD costs per individual are highest in later stages, the greater number of early-stage CKD cases means the majority of the cost burden is located among early-stage cases. Primary and secondary prevention strategies are likely key to reducing costs.

**Supplementary Information:**

The online version contains supplementary material available at 10.1186/s12913-024-11953-6.

## Introduction

Chronic Kidney Disease (CKD) is estimated to affect over 1.7 million people in Australia over 18 years of age, resulting in nearly 20,000 deaths each year [1]. As Australia’s population ages and the burden of non-communicable diseases continues to grow, the cost of managing CKD has emerged as a critical concern for the healthcare system and society at large.

Australia’s healthcare system is primarily publicly funded, including hospital services, general practitioner (GP) visits, medication and a significant proportion of medical tests and specialist care. Co-payments are however common particularly for medication and GP visits. A two-tier system with a private health insurance market exists for inpatient care. Public funding covers renal replacement therapies such as dialysis.

The significant cost of renal replacement therapies such as dialysis for the small proportion of end-stage kidney disease patients is well documented, with dialysis costs averaging $85,000AUD per patient-year [2]. Less well documented are the costs of early-stage CKD which may be significant given increasing prevalence [3]. Understanding the full economic impact of CKD is essential for policymakers to allocate resources effectively, implement targeted interventions, and design healthcare strategies that not only provide quality care but minimise the economic burden on individuals and society.

Several studies have previously examined the cost of CKD in Australia; results are summarised in Table 1. Studies varied in their perspective, methodology, stages of CKD included and cost components under analysis. Studies focusing on early-stage CKD were limited, with most studies focused solely on the cost of end-stage kidney disease [2, 4, 5]. Studies including all CKD stages typically used a ‘top-down’ costing approach where government healthcare expenditure was apportioned amongst diseases [1, 6]. The key exception to this used survey data from the AusDiab study, which allowed bottom-up stratification of costs by CKD stage [7]. This study found healthcare costs 50% higher for those with early-stage CKD when compared to a control population and suggested a significant burden of CKD costs lay in the early stages of the disease. However, the underlying data for this study was collected in 2004/05. The last 20 years have seen significant changes in the healthcare system. An aging population with high rates of comorbidities have contributed to increased healthcare utilisation costs [8], while advances in medical technology and treatments, while improving patient outcomes, come with substantial costs. Challenges in workforce recruitment and retention, particularly within specialised areas such as nephrology [9], have posed additional challenges to managing CKD cost-effectively. Up-to-date estimates are needed to support effective decision-making.

**Table 1:** Previous studies on the cost of CKD in Australia

Paper	Data Source	Perspective	Stages	Base Year	Cost components	Costs
CAS2006 [4]	Registry data	Government	ESKD	2006	Dialysis/transplant costs	$560 m per year
ESS2013 [10]	Survey	Patient	3–5	2011	Medical costs, home assistance, equipment, home modifications	$3,628 per patient-year
WYL2015 [7]	Survey	Societal	1–5	2012	Health (hospital, ED, GP, specialist, medication), non-health (supported care, transport, home service), non-health government (pensions, benefits)	$2,594 per patient-year attributed to CKD. $1,824 in direct healthcare costs Total attributable direct healthcare cost per year $2.5 billion Health costs attributable to CKD: Stages 1/2: $890; Stage 3: $1,660
GOR2019 [2]	Government Data	Government	ESKD	2017	Dialysis costs	$85,919 per patient-year for urban patients. > $120 K for remote patients
STA2021 [5]	Government Data	Government	ESKD- transplant	2018	Immunosuppressant drugs	$71 million per year
SCH2023 [11]	Survey	Patient	3–5	2021	Transport, out of pocket medication, specialist, GP ambulance costs, home care, accommodation	$5,056 per year (rural patients only)
DEL2023 [6]	Government Data	Societal	All	2021	Health system (hospital, outpatient, ED, imaging, pathology, specialists, GPs, medication), productivity, transfers, and other	$9.9 billion total cost (healthcare costs $2.3 billion)
AIHW2023 [1]	Government Data	Government	All	2021–22	Hospital, ED, outpatient, medication, specialist	$1.9 billion per year; $2,326 per person (2018–19 AUD)

*ESKD* End-stage kidney disease, *ED* Emergency department, *GP* General practitioner, *CKD* Chronic kidney disease

This study aimed to provide accurate and up-to-date information on the direct healthcare cost of CKD using a bottom-up approach to allow a detailed understanding of the specific components contributing to the cost of CKD. To do this, we utilise novel data from large-scale administrative linked databases.

## Methods

### Data

This study utilised the CKD.WA dataset, which contains unit-level data from four pathology providers in Western Australia (WA), providing almost complete coverage of pathology testing for WA. Pathology providers included the state-run PathWest and the three main private providers; Western Diagnostics, Clinipath and Australian Clinical Laboratories. The cohort included all Western Australians over 18 years of age with a serum creatinine test recorded between 2006 to 2022. For these individuals, additional blood test results were available, including urine albumin creatinine ratio (uACR), although not from all pathology providers. In addition, all these individuals’ hospital, emergency and mortality records from WA were linked (from the Hospital Morbidity Data Collection, Emergency Department Data Collection and Death Registrations collections respectively), providing secondary care history [12]. The CKD.WA dataset was used to identify prevalence, staging and derive hospital, emergency and pathology costs.

A separate dataset containing extracts from clinical information systems of 39 general practices in WA was used to derive medication and general practitioner (GP) costs for CKD patients. This dataset, a sample of the MedicineInsight dataset for Western Australia [13, 14], included information on diagnoses, pathology results, prescriptions, and billing information for over 500,000 patients. MedicineInsight aims to contain a representative sample of general practices in Australia, and the 39 practices in our sample include practices across socioeconomic areas (20 of 39 practices in the lower socioeconomic half of the state) and geographic regions (8 practices in regional areas, 4 in remote areas).

### Approach

This study used a bottom-up costing approach based on the use of administrative linked data for individuals (and matched controls). Direct healthcare costs were calculated using a government perspective; patient costs were not included. Costs calculated included hospital admissions, emergency presentations, medications, general practice visits, pathology tests, dialysis treatments and outpatient services. Costs were calculated for 2019, the most recent year for which full pathology data was available.

Individuals’ CKD staging was derived from pathology datasets. Individuals could be in more than one stage during the year; where individuals were in more than one stage, their costs were calculated separately for each time period.

Cases were matched to controls on 5-year age band, sex, indigenous status, remoteness (major cities, regional or remote) and evidence of diabetes. Diabetes was defined based on pathology results: evidence of two separate HbA1c results > 6.5% or two separate fasting plasma glucose tests > 7.0 mmol/L. Controls were individuals within the CKD.WA dataset for whom we had their pathology test results and who had no evidence of CKD. Cases were matched 1:1; where a match could not be found, conditions were relaxed in the following order: remoteness, then sex, then indigenous status. Each matched control was apportioned the same length of follow-up time as their partnered case, with derived costs proportionally adjusted to match the length of time.

Costs for each CKD patient were compared to the costs of their matched control. The difference in costs between case and control for each cost component (e.g. cost of any hospital admissions) was taken to be the cost that could be attributed to CKD.

Costs were expressed in 2023 Australian dollars. Where available, unit costs for 2019 were used and then inflated to 2023 dollars by applying appropriate price indices. Where unavailable, unit costs for 2023 were used.

Ethical approval for this project was received from the WA Health Human Research Ethics Committee (HREC) (RGS1183) and Curtin University HREC (HRE2019-0303 & HRE2019-0619).

### CKD definition and staging

Individuals within the CKD.WA dataset were identified as having CKD through either (a) at least two pathology records over 90 days apart with an eGFR < 60 mL/min/1.73m^2^,, with all other eGFR tests occurring within this window also less than 60 mL/min/1.73m^2^,; (b) at least two pathology records over 90 days apart with a urine albumin-creatinine ratio (uACR) ≥ 2.5 mg/mmol for males or uACR ≥ 3.5 mg/mmol for females twice, with all other uACR tests occurring within this window over these cutoffs (i.e. consistently high uACR for over 90 days); EGFR was calculated from serum creatinine using the CKD-EPI formula [15].

CKD staging was based on the standard KDIGO staging system, where uACR was used in combination with creatinine to identify individuals with CKD stages 1 and 2 [16]. Where the two creatinine results over 90 days apart suggested different CKD staging, the lowest (least severe) stage was used. In addition to pathology results, individuals with CKD with a hospital ICD code indicating dialysis (Z49) were classified as Stage 5. Data limitations meant we were unable to accurately identify all individuals undergoing dialysis (in particular, those undergoing home-based dialysis); as such, dialysis patients were included in Stage 5.

A longitudinal history of an individual’s CKD stage was created to identify their CKD staging throughout 2019. This could be defined through records in 2019 or from previous years. An individual’s CKD stage could change throughout 2019; however, CKD staging could only progress, with the CKD stage remaining the same even if eGFR results improved.

Individuals were considered lost to follow-up and excluded if there were no pathology, hospital, emergency or mortality records occurring in 2019 and none occurring after (many datasets continued to 2022), even if prior records identified them as having CKD. Mortality data was used to determine the end of follow-up of individuals who died in 2019.

### Costing

Hospital separation costs were based on the Australian-Refined Diagnosis-Related Groups (AR-DRGs) code captured for each separation. AR-DRG codes were mapped to unit costs for 2019. Hospital admissions for dialysis were not included in a patient’s total hospital costs but were costed in a separate category. Emergency presentation costs were based on National Hospital Cost Data Collection average costs, with defined costs stratified by episode end status.

Medication and GP costs were sourced from an extract from the clinical information systems of nearly 40 general practices in WA. Patients from this dataset had their CKD stage determined using diagnosis information and creatinine pathology results using the staging method described above. GP costs were derived from the recorded Medicare Benefits Schedule (MBS) item numbers for each patient for 2019. Prescription costs were derived from all prescribed medications in 2019. For each medicine prescribed, the cost for all repeats was calculated and added to the 2019 total; while some of these costs would likely occur in 2020, it was assumed these would be equivalent to the uncosted medicines prescribed in 2018 that would be filled in 2019. Medication costs included the cost of each prescription along with the dispensing fee, handling fee and Pharmaceutical Benefits Scheme (PBS) Safety Net recording fee. The cost of a medication borne by the government depended on an individual patient’s concessional status and whether they had reached the PBS safety-net threshold (a set amount spent on medications per calendar year, after which patient medications on the PBS are provided at reduced cost or free of charge to the patient for the rest of the year. An average cost per medication was calculated and used, derived by taking a weighted average of the different prices of the medication (depending on concessional status/safety net) by how often the particular medication was prescribed to concessional patients/under the safety net in Australia (this data was publicly available [17]).

Once individuals within the GP dataset had their CKD stage, GP and medication costs calculated, statistical models were developed to predict GP and medication costs. These models were then applied to the CKD.WA dataset to derive GP and medication cost estimates for all individuals with CKD in WA. Generalised linear modelling was used to estimate costs. Several varieties of this model were tested, with the final chosen model using a gamma distribution with a log-link function. Factors used to predict costs included CKD Stage (No CKD, Stage 1, Stage 2, Stage 3a, Stage 3b, Stage 4, Stage 5), sex, age (5-year age bands), indigenous status and remoteness (major cities, regional or remote). The final model was chosen by comparing models for goodness of fit using the Akaike Information Criterion and the Bayesian Information Criterion. In addition, predicted results were compared to actual values for each dependent variable to ensure appropriateness of fit.

While pathology data was provided from all WA providers for the CKD.WA dataset, the range of provided data differed between providers. Western Diagnostics provided the most complete extract, including the widest range of tests. To provide more accurate estimates of pathology costs, pathology costs from patients who used Western Diagnostics predominantly were modelled and then applied across the whole CKD.WA dataset. Individuals who had > 95% of community pathology tests with Western Diagnostics were included in the model; predictors and the final model type were the same as with medication and GP cost modelling.

Pathology costs included the MBS collection and bulk billing fees plus the cost of the three most expensive MBS items within a collection (the ‘coning’ rule [18]). Pathology tests costed included urea, creatinine and electrolytes, full blood count, blood lipids, blood sugars including HbA1c, thyroid function tests, iron studies, liver function tests, troponin, Vitamin D, calcium, rheumatoid factor, complements, bone markers, parathyroid hormone and a range of antibody tests. Inpatient pathology testing was included as part of hospital costs.

Dialysis costs included both in-hospital or satellite haemodialysis, which were costed as hospital admissions using AR-DRGs as above, and home-based haemodialysis and peritoneal dialysis. Home-based haemodialysis and peritoneal dialysis were charged as Tier 2 Non-Admitted Patient Services. No individual-level costing was available for home-based dialysis, and so top-down costing was applied. The total cost and number of home-based dialysis services were derived from government reports [19, 20]. Home-based dialysis costs were assigned to Stage 5 CKD.

A top-down approach was used for attributing the cost of outpatient services. The total cost of nephrologist-led and nurse-practitioner-led renal clinics was sourced from government reports [19, 20]. Costs were distributed by CKD stage based on frequency estimates of outpatient presentations by Western Australian nephrologists.

### Extrapolating early-stage CKD prevalence

While kidney screening using creatinine tests is common in the population, initial analysis identified a significantly lower proportion of individuals who received uACR testing. With limited symptoms experienced during early-stage CKD and the requirement for uACR testing for diagnosis, a level of under-diagnosis for early-stage CKD is likely.

To account for this and estimate the true number of Stage 1–2 patients, figures from the *Australian Health Survey 2011–12 *^*21*^ were used alongside CKD.WA data. This survey involved testing and recording biomedical results, including creatinine and uACR from a random sample of the population, allowing us to derive the proportion of individuals with eGFR 60–89 mL/min/1.73 who also had albuminuria and therefore CKD Stage 2 (9.7%) and likewise the proportion of individuals with eGFR ≥ 90 mL/min/1.73m^2^ but albuminuria (and therefore CKD Stage 1; 5.7%). The *Australian Health Survey* conducted only a single measurement of uACR. To account for chronicity, these proportions were multiplied by the proportion of individuals with a recorded measurement of albuminuria who still had albuminuria at > 90 days, using the CKD.WA dataset.

### Extrapolating costs to the whole of Australia

To derive an Australia-wide cost figure, the average attributable CKD cost per person and the number of cases were calculated for each combination of stage, sex, indigenous status, 5-year age bands, and remoteness. The number of cases in each combination was then divided by the number of people in WA with this combination and multiplied by the number of people in Australia for this combination, to derive estimates of the number of individuals across Australia in each combination with CKD. These counts were then multiplied by the average attributable cost of CKD per person for that stage and summed to identify the total cost Australia-wide for all stage CKD. This allowed cost estimates to take into account differences in indigenous status, remoteness, age and sex distributions between Western Australia and the rest of Australia.

## Results

There were 114,899 individuals in WA with known CKD at some stage in 2019. Table 2 shows basic demographic information for the cohort by CKD stage. Individuals could be in more than one CKD stage during the year. There were less individuals with Stage 1–2 CKD as compared with Stage 3, due to lower rates of testing for uACR (required for diagnosing Stages 1–2). Individuals in Stages 1–2 were more likely to be in remote areas, indigenous and to have diabetes than their counterparts in Stages 3–4. Individuals in Stage 5 were also more likely to be indigenous and remote. Median age increased through the stages, from 53 years in Stage 1 CKD to 79 and 82 years in Stages 3 and 4, before dropping in Stage 5 to 64 years of age.

**Table 2:** Demographic information by CKD stage

CKD Stages						
	Stage 1	Stage 2	Stage 3a	Stage 3b	Stage 4	Stage 5
Number of individuals	8,338	9,179	66,524	26,573	7,879	4,031
Person years	7,195	7,786	57,973	22,376	6,205	3,517
*Sex*						
Male	5,022 (60%)	6,078 (66%)	30,772 (46%)	12,053 (45%)	3,898 (49%)	2,360 (59%)
Female	3,316 (40%)	3,101 (34%)	35,752 (54%)	14,520 (55%)	3,981 (51%)	1,671 (42%)
*Remoteness*						
Major Cities	4,589 (55%)	6,045 (66%)	50,208 (76%)	20,194 (76%)	5,900 (75%)	2,794 (69%)
Regional	1,323 (16%)	1,594 (17%)	13,353 (20%)	5,162 (19%)	1,488 (19%)	670 (17%)
Remote	2,360 (28%)	1,485 (16%)	2,912 (4%)	1,199 (5%)	487 (6%)	561 (14%)
*Age category*						
< 50	3,276 (39%)	782 (9%)	1,133 (2%)	472 (2%)	321 (5%)	764 (19%)
50–59	2,382 (29%)	1,467 (16%)	2,898 (4%)	741 (3%)	359 (5%)	788 (20%)
60–69	2,092 (25%)	2,631 (29%)	10,035 (15%)	2,362 (9%)	805 (10%)	923 (23%)
70–79	553 (7%)	2,959 (32%)	23,295 (35%)	6,940 (26%)	1,756 (22%)	847 (21%)
80–89	35 (0%)	1,206 (13%)	22,458 (34%)	10,880 (41%)	2,911 (37%)	558 (14%)
90 +	0	134 (1%)	6,705 (10%)	5,178 (19%)	1,727 (22%)	151 (4%)
*Indigenous*	2,648 (32%)	1,608 (18%)	1,532 (2%)	747 (3%)	429 (5%)	785 (19%)
*Has Diabetes*	3,641 (44%)	4,491 (49%)	7,919 (12%)	4,112 (15%)	1,568 (20%)	598 (15%)

In total, diagnosed CKD in WA cost the government $704 million in 2019. This included $24 million for Stage 1, $32 million for Stage 2, $209 million for Stage 3a, $147 million for Stage 3b, $71 million for Stage 4 and $220 million for Stage 5. Costs in Stage 3 made up half of total costs, while the cost of CKD Stage 5 was 31% of the total cost of CKD. Having CKD increased an individual’s healthcare costs on average by 75%; for Stages 1–3 it increased health costs by 50%, for Stage 4 CKD doubled health costs, and for Stage 5 CKD increased costs by nearly 10 times.

Costs attributable to CKD by cost component are shown in Table 3. More than half of costs were related to non-dialysis hospital admissions; other significant costs were dialysis (including hospital, satellite and home-based dialysis) and medication costs.

**Table 3:** Total costs attributable to CKD by cost component

	Total Costs Attributable to CKD (million $)	%
Emergency Presentations	46	7%
Hospital admissions (excluding dialysis)	396	56%
Medication	84	12%
Primary Care	31	4%
Community Pathology Testing	7	1%
Dialysis	130	18%
Outpatient Services	9	1%

Average costs by person-year for each stage and cost component are shown in Table 4. Costs for all components generally increased steadily as CKD progressed. A notable exception for this was medication costs which were much higher in Stages 1–2 as compared with Stage 3. Closer examination suggested this was primarily due to the higher prevalence of diabetes in early-stage CKD – while diabetes was controlled for, individuals who had both CKD and diabetes had higher medication usage and associated costs than those with CKD or diabetes alone. Not all Stage 5 patients had dialysis costs resulting in a reduced average dialysis cost of $36,982.

**Table 4:** Attributable average cost per person-year by stage and cost component

	Stage 1 ($)	Stage 2 ($)	Stage 3a ($)	Stage 3b ($)	Stage 4 ($)	Stage 5 ($)
Emergency Costs	148 (4%)	255 (6%)	269 (7%)	550 (8%)	1,028 (9%)	2,454 (4%)
Hospital Costs (no dialysis)	1,515 (45%)	2,255 (55%)	2,470 (68%)	4,592 (70%)	8,400 (73%)	19,723 (32%)
Medication Costs	1,374 (41%)	1,172 (28%)	577 (16%)	831 (13%)	1,121 (10%)	1,780 (3%)
Primary Care	315 (9%)	401 (10%)	230 (6%)	347 (5%)	463 (4%)	474 1%)
Pathology Testing	14 (0%)	31 (1%)	47 (1%)	96 (1%)	168 (1%)	203 (0%)
Dialysis Costs	0 (0%)	0 (0%)	0 (0%)	0 (0%)	0 (0%)	36,982 (59%)
Outpatient Services	0 (0%)	0 (0%)	15 (0%)	156 (2%)	274 (2%)	942 (2%)
**Total**	**3,367**	**4,114**	**3,607**	**6,572**	**11,456**	**62,558**

Increases in pathology and primary care costs by stage were primarily a function of increased service use (i.e. more blood tests/GP visits) rather than using different or more expensive services. Increased medication costs in early-stage CKD (as compared to non-CKD) were largely driven by greater rates of prescriptions for statins, angiotensin converting enzyme inhibitors and other diabetes medications; higher rates of prescriptions for angiotensin receptor blockers and cardiovascular medications drove increased costs as CKD progressed. Drivers of hospital and emergency costs were more mixed and included increases in admissions for cardiovascular, renal and endocrine-related conditions, but also other conditions less related to CKD, including cancer hospitalisations, injury hospitalisations and respiratory hospitalisations. No difference was seen for mental health hospitalisations.

### Identifying and costing undiagnosed CKD

The majority of the adult Western Australian population had a creatinine test available in our dataset, including 100% of those over 65 years of age and 93% of those over 50 years of age. For uACR, only 24% of Western Australian adults had a test result within our dataset. Using figures from the *Australian Health Survey * [21] along with derived estimates of the proportion of individuals with albuminuria which lasted greater than 90 days (66%), the estimated number of individuals with CKD Stages 1 and 2 is 40,363 and 33,482 respectively, suggesting the number of undiagnosed CKD Stage 1 and 2 patients is 32,026 and 24,303 respectively. Using the same attributable person-cost for these individuals, this results in an additional Stage 1 and Stage 2 cost of $108 million and $100 million, increasing the total cost to $912 million.

### Estimating the cost to Australia

Extrapolating the results of WA to the broader Australian population, we estimate the cost attributable to CKD in 2019 in Australia at $8.33 billion, including $1.6 billion for early-stage CKD (Stages 1 & 2), $3.55 billion for Stage 3 CKD, and $0.91 billion and $2.27 billion for Stages 4 and 5, respectively.

## Discussion

Chronic kidney disease is responsible for significant increases in health costs, costing Australia an estimated $8.33 billion in 2019 just in direct healthcare costs. This is significantly higher than previous comparable estimates, including by the Australian Institute of Health and Welfare (AIHW) ($1.8 billion) [1], Deloitte ($2.3 billion) [6] and a study by Wyld et al. [7] ($2.5 billion), even after adjusting to 2023 dollars. At the per-person level, the cost of CKD rose from $3,367 per person-year for CKD Stage 1 to $6,572 for CKD Stage 3b to $62,558 per person-year for Stage 5. Our figures for both early and later-stage CKD are greater than those of Wyld et al. [7], despite measuring most of the same cost components. Some of this difference may be explained by changes in treatment over this time, such as the introduction of medications such as SGLT2 inhibitors. While improved preventative treatments may lead to slightly higher costs in early CKD stages, we should ultimately expect reduced costs because of these treatments, primarily from reduced hospitalisations. Other reasons for the difference between our and Wyld’s estimates may be due to differences in the data collection, with the AusDiab survey less accurate at capturing full cost information, or a level of response bias where more educated, health literate responders engaged with the AusDiab survey, who may be more engaged in preventative health strategies, reducing comorbidities.

While per-person costs generally increased as CKD stage increased, the notable exception to this was Stage 2 costs, which were slightly higher than Stage 3a costs. These higher costs were driven primarily by the much higher rates of diabetes in both Stage 1 and Stage 2. While diabetes was controlled for in this study (by comparing patients with CKD and diabetes to those controls with diabetes), the combination of both conditions led to increased costs, in particular medication costs. The high rates of diabetes in our early-stage CKD cases represents a sampling issue, with individuals with diabetes more likely to receive uACR testing and therefore be diagnosed as early-stage CKD, effectively biasing our costs upwards. However results from other studies, including the AusDiab study and data from the NHANES study in the US [22] have also shown higher diabetes rates similar to our results in early-stage CKD, when measuring a random sample of the population (i.e. without likely selection bias).

While the highest per person costs were found in those with Stage 5 CKD, the greater number of individuals in Stages 1–3 CKD meant that it was these stages which made up the majority of the costs of CKD. This highlights the importance of focusing on prevention strategies to reduce the risk of CKD progression to end-stage kidney disease.

Challenges exist in attempting to attribute healthcare costs to specific conditions. A top-down approach whereby total healthcare expenditure is split based on primary cause will underestimate costs, particularly for conditions such as CKD, as it will not consider the significant non-renal comorbidities CKD is known to cause, most significantly cardiovascular disease. AIHW used this top-down approach to reach their $1.8 billion total healthcare cost for CKD in Australia, which in turn was used as the basis for Deloitte’s $2.3 billion estimation. These costs are almost certainly underestimates of the true healthcare costs of CKD in Australia.

An alternate approach, as carried out here and in previous work by Wyld et. al [7]., is to calculate the total healthcare expenditure of individuals with CKD. This can then be compared to the total healthcare expenditure of similar individuals without CKD, with the difference between them assumed to be the cost that can be attributed to CKD. While these costs will be more accurate than the top-down approach, there remain limitations with this methodology as well. It can be expected that, on average, our CKD cases will have higher rates of CKD risk factors (obesity, diabetes, hypertension) than our controls simply given the fact that they have CKD; our cases may also have higher costs due to comorbidities caused by these risk factors *independent of* CKD. Our analysis was able to account for and control for any additional cost of diabetes by comparing the cost of CKD patients with diabetes with the cost of non-CKD patients with diabetes; however, we were not able to do this for every condition. There was some evidence that our CKD cohort was, in general, ‘sicker’ than our controls, with increased hospitalisations found in our CKD cohort across many conditions not directly related to CKD. As such, the methodology used here may overestimate the true cost attributable to CKD.

This study faced several other limitations. GP, medication and pathology costs were unavailable at a unit-record level and had to be modelled. Data used to model pathology costs included only a specific set of common blood tests and did not include the larger pool of less common and more expensive pathology tests, potentially biasing costs downwards. Medication costs were derived from GP data and may not include all medications prescribed by specialists such as nephrologists (these would only be recorded if/when the GP provides a repeat script). Aboriginal medical services were not included within our GP dataset, and to the extend aboriginal patients who attended these services differed from those who attended other general practitioners, medication and GP costs for this cohort may be inaccurate. Several healthcare costs were not included, such as medical imaging, allied health, specialist consults outside of renal clinics, non-renal outpatient services and ambulance services. This study looked only at the cost to government and did not include costs incurred by the patient. Recent studies have suggested this cost is significant, with costs in the thousands per year [10], leading to significant financial distress for patients [11]. The study also did not look at the broader societal implications of CKD, which are significant [6, 23]. The study could not accurately distinguish between those in Stage 5 undergoing dialysis and those who were not, and these categories had to be collapsed together. No separate costing could be given for transplant patients given limitations in the data; transplant patients were instead classified based on eGFR. Our GP extract had limited information on medication costs for transplant patients, and it is expected these will be underestimated. This project attempted to both count and cost the ‘undiagnosed’ early-stage CKD patients. These counts and costs rely on a series of assumptions regarding the extent of undiagnosed CKD, and the expected cost of these patients. Significantly more caution should be given regarding the accuracy of costs relating to undiagnosed CKD.

The estimated substantial financial cost of CKD underscores the profound societal and economic impact of this chronic condition. Cost is but one key factor to consider in determining health service decision making, and any comprehensive analysis must balance this with an understanding of health outcomes and patient experiences, along with broader societal factors. Notwithstanding this, these findings suggest prevention through focusing on early risk factors appears key to reducing costs. By addressing CKD comprehensively, we can not only alleviate the financial burden on the healthcare system but also improve the health and quality of life of many.

## Supplementary Information

Supplementary Material 1.

## Data Availability

The datasets used here are not publicly available due to privacy considerations. Researchers may apply to the relevant data custodians for access.
